# Radiotherapy-Induced Anti-Tumor Immunity Contributes to the Therapeutic Efficacy of Irradiation and Can Be Augmented by CTLA-4 Blockade in a Mouse Model

**DOI:** 10.1371/journal.pone.0092572

**Published:** 2014-03-31

**Authors:** Yuya Yoshimoto, Yoshiyuki Suzuki, Kousaku Mimura, Ken Ando, Takahiro Oike, Hiro Sato, Noriyuki Okonogi, Takanori Maruyama, Shinichiro Izawa, Shin-ei Noda, Hideki Fujii, Koji Kono, Takashi Nakano

**Affiliations:** 1 Department of Radiation Oncology, Gunma University Graduate School of Medicine, Maebashi, Japan; 2 Department of Surgery, National University of Singapore, Singapore, Singapore; 3 Cancer Science Institute of Singapore, National University of Singapore, Singapore, Singapore; 4 First Department of Surgery, University of Yamanashi, Yamanashi, Japan; Mie University Graduate School of Medicine, Japan

## Abstract

**Purpose:**

There is growing evidence that tumor-specific immune responses play an important role in anti-cancer therapy, including radiotherapy. Using mouse tumor models we demonstrate that irradiation-induced anti-tumor immunity is essential for the therapeutic efficacy of irradiation and can be augmented by modulation of cytotoxic T lymphocyte (CTL) activity.

**Methods and Materials:**

C57BL/6 mice, syngeneic EL4 lymphoma cells, and Lewis lung carcinoma (LL/C) cells were used. Cells were injected into the right femurs of mice. Ten days after inoculation, tumors were treated with 30 Gy of local X-ray irradiation and their growth was subsequently measured. The effect of irradiation on tumor growth delay (TGD) was defined as the time (in days) for tumors to grow to 500 mm^3^ in the treated group minus that of the untreated group. Cytokine production and serum antibodies were measured by ELISA and flow cytometry.

**Results:**

In the EL4 tumor model, tumors were locally controlled by X-ray irradiation and re-introduced EL4 cells were completely rejected. Mouse EL4-specific systemic immunity was confirmed by splenocyte cytokine production and detection of tumor-specific IgG1 antibodies. In the LL/C tumor model, X-ray irradiation also significantly delayed tumor growth (TGD: 15.4 days) and prolonged median survival time (MST) to 59 days (versus 28 days in the non-irradiated group). CD8(+) cell depletion using an anti-CD8 antibody significantly decreased the therapeutic efficacy of irradiation (TGD, 8.7 days; MST, 49 days). Next, we examined whether T cell modulation affected the efficacy of radiotherapy. An anti-CTLA-4 antibody significantly increased the anti-tumor activity of radiotherapy (TGD was prolonged from 13.1 to 19.5 days), while anti-FR4 and anti-GITR antibodies did not affect efficacy.

**Conclusions:**

Our results indicate that tumor-specific immune responses play an important role in the therapeutic efficacy of irradiation. Immunomodulation, including CTLA-4 blockade, may be a promising treatment in combination with radiotherapy.

## Introduction

Recently, several reports showed that radiotherapy and anti-tumor immunity are closely associated. We recently demonstrated that tumor antigen-specific T cell responses can be induced in esophageal cancer patients during and after chemoradiotherapy [1]. We detected specific T cells recognizing antigen-derived peptides in a HLA class I-restricted manner using ELISPOT analysis of patient samples [1]. Clinically, the abscopal effect is a well-known but rare phenomenon in which local radiotherapy is associated with the regression of a metastatic tumor located at a distance from the irradiated site. This effect is thought to be mediated by activation of anti-tumor immunity. Postow *et al.* reported a case of the abscopal effect in a patient with melanoma treated with radiotherapy and ipilimumab, an antagonistic antibody against cytotoxic T-lymphocyte-associated antigen 4 (CTLA-4). In this case, disease resolution after radiotherapy was associated with a specific antibody response [2]. Demaria *et al.* used a mouse syngeneic mammary carcinoma model to show that abscopal effects result from irradiation-activated anti-tumor immunity [3]. Taken together, these observations indicate that local radiotherapy can induce systemic tumor-specific immune responses.

The molecular mechanisms that mediate anti-tumor immunity, in terms of irradiation-induced immunogenic tumor cell death and its impact on the prognosis of cancer patients, have also been investigated. Apetoh *et al.* reported that activation of tumor antigen-specific T cell responses involve the secretion of high-mobility-group box 1 (HMGB1) alarmin protein from dying tumor cells and the action of HMGB1 on Toll-like receptor 4 (TLR4)-expressing dendritic cells [4]. This pathway and activated anti-tumor immunity play important roles in human cancer, as patients with breast cancer who carry a *TLR4* loss-of-function allele relapse more quickly after radiotherapy and chemotherapy. HMGB1 may also be a prognostic factor; its up-regulation within the tumor microenvironment is positively correlated with esophageal cancer patient survival after chemoradiotherapy [1], although the significance of HMGB1 is still controversial. Thus, radiotherapy-induced immune responses may contribute to the therapeutic efficacy of irradiation. However, the immune system does not always exert robust responses, such as the abscopal effect, suggesting the existence of suppressor mechanisms. Regulatory T (Treg) cells mediate one of the most important mechanisms for suppression of effector T cell responses. Treg cells are characterized as CD4(+)CD25(+)FoxP3(+) and have a critical role in the maintenance of immunological self-tolerance [5]. Treg cells suppress effector cells by co-localizing Treg and effector cells with antigen presenting cells [6], and also by inhibiting the release of cytolytic granules from effector T cells [7]. Cancer patients have increased levels of Treg cells, resulting in poor immune responses to tumors. Thus, Treg cell depletion may be an effective cancer treatment [5].

In this study, we used mouse models and immunomodulatory antibodies to test whether irradiation-induced anti-tumor responses are essential for the efficacy of irradiation and whether this effect can be augmented by T cell modulation.

## Materials and Methods

### Mice, cell lines and antibodies

C57BL/6 mice and BALB/c-*nu*/*nu* mice were purchased from Japan SLC (Shizuoka, Japan). Mice were bred and maintained under specific-pathogen-free conditions. C57BL/6 syngeneic Lewis lung carcinoma cells (LL/C; mouse lung squamous carcinoma) were purchased from American Type Culture Collection (Manassas, VA). Cells were cultured in RPMI 1640 supplemented with 5% fetal calf serum (FCS), 50 U/ml penicillin, and 2 mM L-glutamine. RPMI 1640 and FCS were purchased from Invitrogen (Carlsbad, CA) and penicillin was purchased from Sigma-Aldrich (St. Louis, MO). All procedures for the care and treatment of animals were performed according to the Japanese Act on the Welfare and Management of Animals and the Guidelines for the Proper Conduct of Animal Experiments issued by the Science Council of Japan. The experimental protocol was approved by the Institutional Committee of Gunma University (09-074). Every effort was made to minimize animal suffering and the number of animals used. Anti-mouse CD8 (clone 53.6.72), anti-mouse folate receptor 4 (FR4; clone TH6), anti-mouse glucocorticoid-induced tumor necrosis factor receptor family-related gene (GITR; clone DTA-1), and anti-mouse CTLA-4 (clone 9H10) antibodies were purchased from eBioscience (San Diego, CA).

### Local irradiation, measurement of tumor volume, and immunomodulatory antibody treatment

C57BL/6 mice and the syngeneic cell lines, LL/C, EL4, and B16 were used. Cells (5×10^5^) were injected into the right/bilateral hind limbs of C57BL/6 mice. When tumors reached 100 mm^3^ in volume, they were exposed to 30 Gy of X-rays at a dose rate of 1.3 Gy/min (TITAN-225S, Shimazu, Japan). Only tumors were irradiated; the rest of the body was shielded by lead. Tumor size was measured using calipers every other day. Tumor volume (V) was calculated using the following formula: V = ab^2^/2 (where a is the long axis diameter and b is the short axis diameter). The effect of irradiation on tumor growth time, the ‘tumor growth delay’ (TGD), was defined as the time in days for tumors to grow to 500 mm^3^ in the treated group minus that in the untreated group. To examine the effect of immunomodulatory antibodies, anti-mouse CD8 antibody (administered intravenously at a dose of 66 μg/mouse on Days 1, 6, and 10), anti-CTLA-4 antibody (administered intraperitoneally at a dose of 200 μg/mouse on Days 1, 4, and 7), anti-FR4 antibody (administered intravenously at a dose of 10 μg/mouse on Days 1, 4, and 7), anti-GITR antibody (administered intratumorally at a dose of 50 μg/mouse on Day 1), and saline (for the non-treated group, administered intravenously on Days 1, 4, and 7) were used.

### ELISA

To evaluate splenocyte function, EL4 tumors borne by C57BL/6 and BALB/c-*nu*/*nu* mice were exposed to 30 Gy of X-rays. Irradiated non-tumor-bearing C57BL/6 and BALB/c-nu/nu mice were used as controls. Spleens were removed at 0, 10, 11, 14, 17, 20 and 24 days after the first tumor cell inoculation and splenocytes (3.0×10^6^/ml) were co-cultured with irradiated EL4 cells (0.3×10^6^/ml) in medium for 24 h. IFN-γ and TNF-α levels in the culture supernatant were then measured using mouse IFN-γ (R&D Systems, Minneapolis) and TNF-α (R&D Systems) ELISA kits, according to the manufacturers’ instructions. To measure HMGB1 concentrations, LL/C and EL4 cells were seeded in culture dishes, exposed to X-rays (Siemens-Asahi Medical Technologies, Tokyo, Japan), and then cultured in RPMI for 48 h. Culture supernatants were collected and HMGB1 concentrations were measured using an ELISA kit (Shinotest, Tokyo, Japan) according to the manufacturer’s instructions.

### Flow cytometric analysis of EL4-specific antibodies

Blood samples were taken from mice described in the previous section 24 days after tumor inoculation. Serum was obtained from blood samples by centrifugation. The serum was diluted 1:5 with phosphate-buffered saline (PBS) and incubated with EL4 cells for 30 min on ice. EL4 cells were then washed and stained with FITC-conjugated anti-mouse IgG1 (eBioscience). Stained cells were analyzed by four-color flow cytometry using a FACSCalibur flow cytometer (BD Biosciences). Data were analyzed using CellQuest™ software (BD Biosciences).

### Statistical analysis

Statistical analysis was performed using two-tailed, unpaired Student’s *t* tests. Error bars represent the standard deviation (SD). For survival curves, statistical analyses were performed using the log-rank (Mantel-cox) test. All statistical analyses were performed using IBM SPSS Statistics software, version 21. A *p* value <0.05 was considered significant.

## Results

### Irradiation of EL4 tumors prevented systemic tumor progression and led to the rejection of re-inoculated tumors in C57BL/6 mice, but not in BALB/c-nu/nu mice

**Figure 1:**
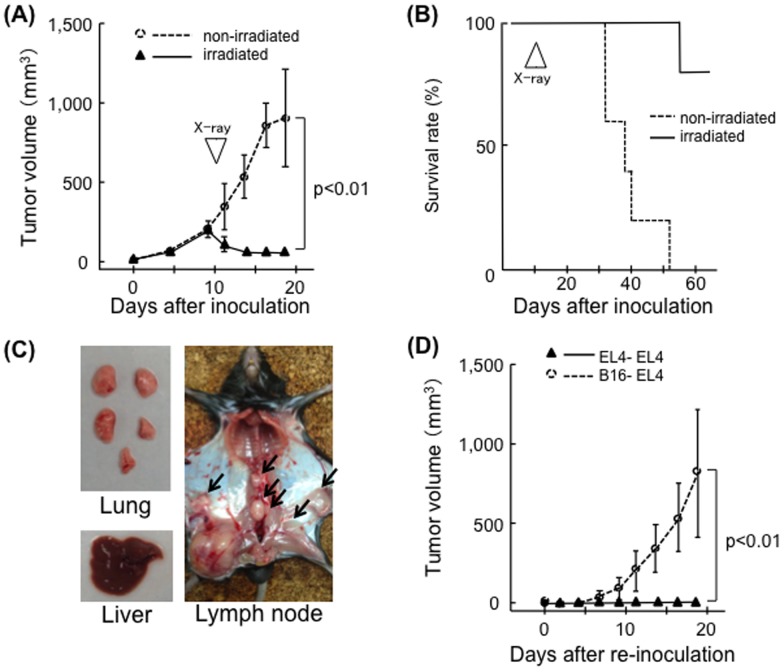
Therapeutic effects of X-ray irradiation on EL4 tumors in C57BL/6 mice. (A) Growth curves of EL4 tumors in the non-treated group (open circles, n = 5) and irradiated group (closed triangles, n = 5). X-ray irradiation was performed on Day 10; bars, SD. (B) Survival curves for EL4-inoculated C57BL/6 mice, the non-treated group (dotted line), and the irradiated group (solid line). (C) Lymph node metastasis (arrows) in a representative mouse 20 days after tumor inoculation. (D) Growth curve of EL4 tumors (second inoculation). EL4 cells were re-inoculated into the left hind limbs of mice 24 days after the first challenge with EL4 cells (closed triangles, n = 5) or B16 cells (open circles, n = 5); bars, S.D.

**Figure 2:**
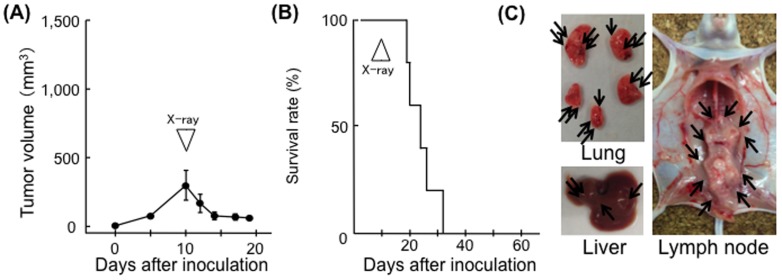
Effect of X-ray irradiation on EL4 tumors in BALB/c-nu/nu mice. (A) Growth curves of EL4 tumors in the irradiated group (n = 5). X-ray irradiation was performed on Day 10; bars, SD. (B) Survival curves for EL4-inoculated BALB/c-*nu/nu* mice. (C) Systemic metastases (arrows) in the internal organs of a representative mouse 20 days after tumor inoculation.

First, the therapeutic effect of X-ray irradiation against EL4 tumors was tested. EL4 tumors were locally controlled by 30 Gy of X-ray irradiation. Irradiation induced a significant decrease in tumor volume (*p*<0.01) and almost all irradiated mice survived (one-fifth died due to marginal recurrence), while non-irradiated mice died within 5–8 weeks of inoculation (Fig. 1A, 1B). Dissections revealed that all mice had significant lymph node metastasis. Liver and lung metastasis was macroscopically less obvious (Fig. 1C). Irradiated mice completely rejected re-inoculated EL4 cells administered 24 days after the first inoculation. EL4 cells were not rejected when B16 melanomas were used in the first inoculation (Fig. 1D). Although irradiation resulted in a reduction in EL4 tumor size in BALB/c-*nu/nu* mice, all animals died within 3 weeks of irradiation (Fig. 2A, 2B). In these cases, systemic tumor metastasis (lymph nodes, lung and liver) was observed (Fig. 2C).

### EL4-specific cellular and humoral immunity was induced in irradiated EL4-bearing C57/BL6 mice

**Figure 3:**
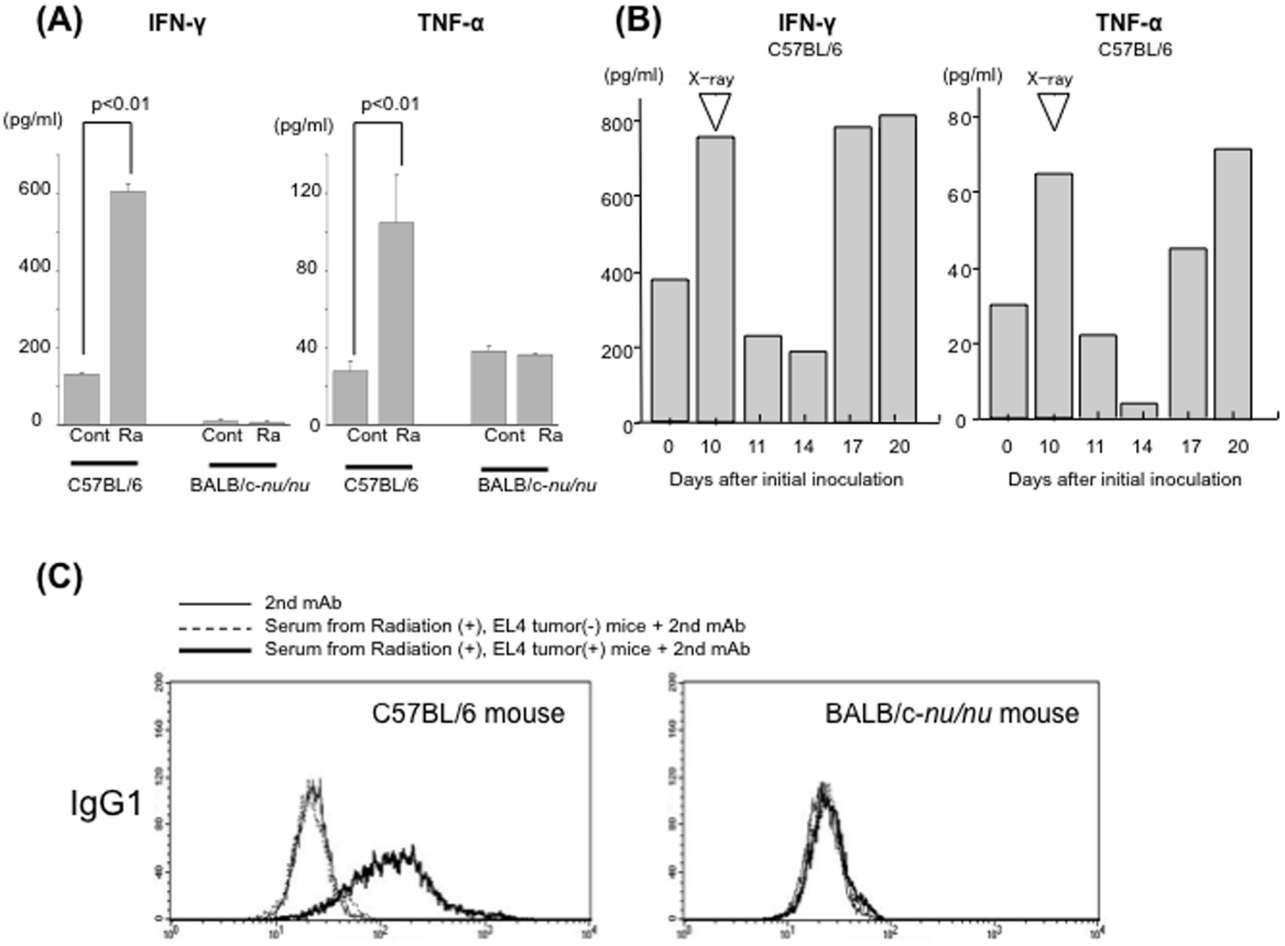
Immune reaction in irradiated EL4 tumor-bearing mice. (A) IFN-γ (left) and TNF-α (right) concentrations in C57BL/6 and BALB-c *nu/nu* mouse splenocyte culture supernatants as measured by ELISA. Mice were sacrificed 24 days after tumor inoculation. Control (cont) splenocytes from non-tumor-bearing irradiated mice; Ra, splenocytes from irradiated EL4 tumor-bearing mice. (B) Mice were sacrificed on the indicated day and splenocytes were cultured for ELISA. Irradiation was performed on Day 10. Day 0, splenocytes from non-tumor-bearing non-irradiated mice; Day 10, tumor-bearing non-irradiated mice; Days 11, 14, 17, 20, tumor-bearing irradiated mice. (C) Antibodies specific for EL4 cells produced by local irradiation of EL4 tumors in C57/BL6 (left) and BALB/c-nu/nu (right) mice.

**Figure 4:**
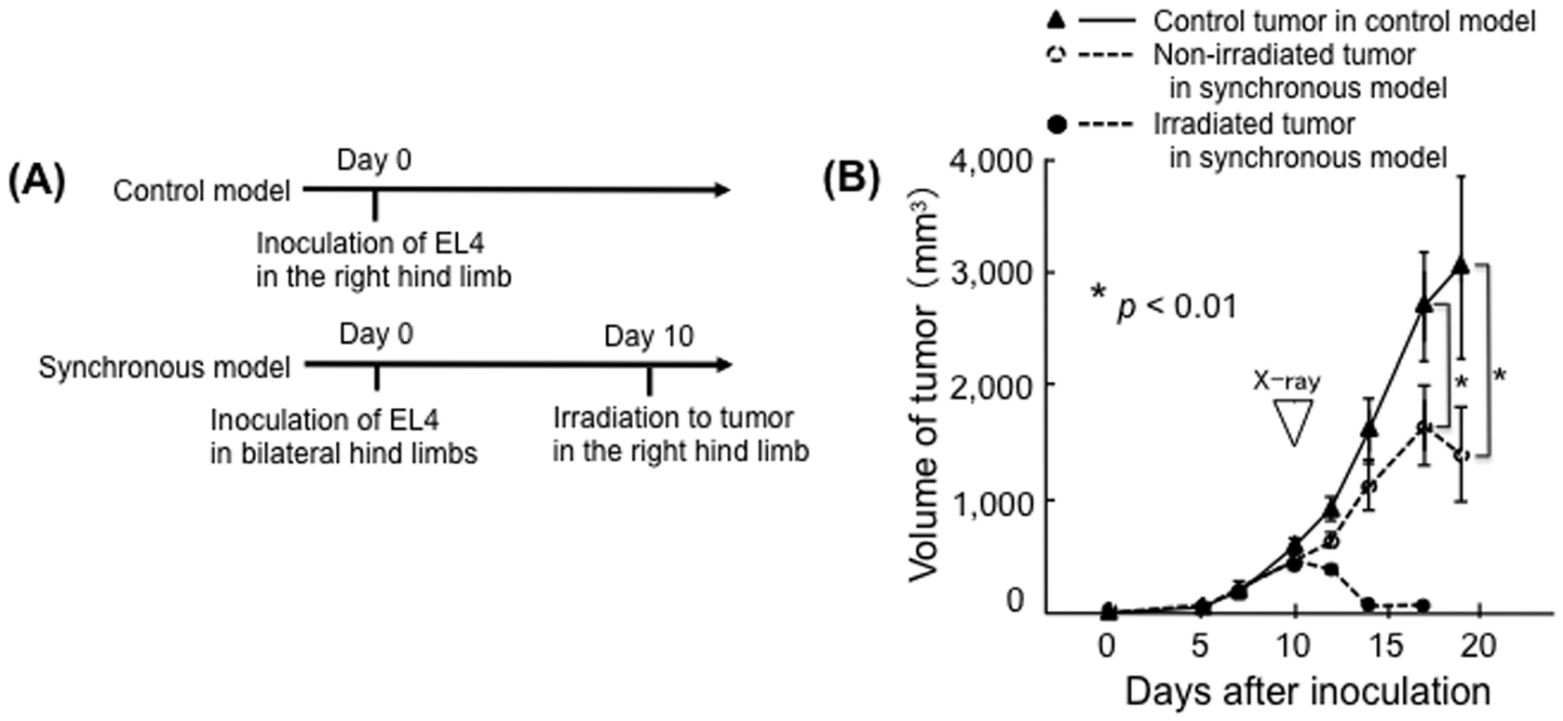
Irradiation of EL4 tumors in C57BL/6 mice results in the abscopal effect. (A) Timing of primary EL4 cell inoculation and tumor irradiation in control and synchronous models. (B) Volume of irradiated tumors in the right hind limbs of synchronous model mice (closed circles, n = 5) and of non-irradiated tumors in the left hind limbs of synchronous model mice (open circles) and the control tumors in control model mice (closed triangles, n = 5); bars, S.D.

To examine cellular immunity in treated mice, splenocyte cytokine production was measured. Splenocytes of irradiated EL4-bearing C57/BL6 mice, but not those of irradiated non-tumor-bearing C57/BL6 mice, produced significant amounts of IFN-γ and TNF-α (Fig. 3A). There was no significant production of these cytokines by splenocytes from either tumor-bearing or non-tumor-bearing nude mice (Fig. 3A). In a time-course study, cytokine production was higher in tumor-bearing mice (Day 10, before irradiation) than in non-tumor-bearing mice (Day 0). After irradiation, cytokine production decreased on Days 11 and 14. However, cytokine production then increased on Days 17 and 20 (Figure 3B). Humoral immunity was examined by flow cytometry. EL4-specific IgG1 was detected in the serum of irradiated EL4-bearing C57/BL6 mice (Fig. 3C). Thus, both cellular and humoral immunity against EL4 tumors were induced in irradiated EL4-bearing C57/BL6 mice. Anti-tumor effects of the immune response against EL4 tumors were also examined *in vivo*. EL4 tumors were inoculated into both the right and left hind limbs; however, only the tumors in the right hind limbs were irradiated. All of the irradiated tumors disappeared within 7 days of irradiation. In addition, irradiation inhibited the growth of the non-irradiated tumors in the left hind limbs; the volumes were significantly smaller than those in control mice on Day 19 post-inoculation (*p*<0.01) (Figure 4). There was no significant difference in tumor growth between the unilaterally inoculated tumors and either of the bilaterally inoculated tumors in non-irradiated mice (Supplemental Figure S1).

### Irradiation delayed LL/C tumor growth in C57BL/6 mice and the therapeutic efficacy of irradiation was reduced by depleting CD8-positive lymphocytes

**Figure 5:**
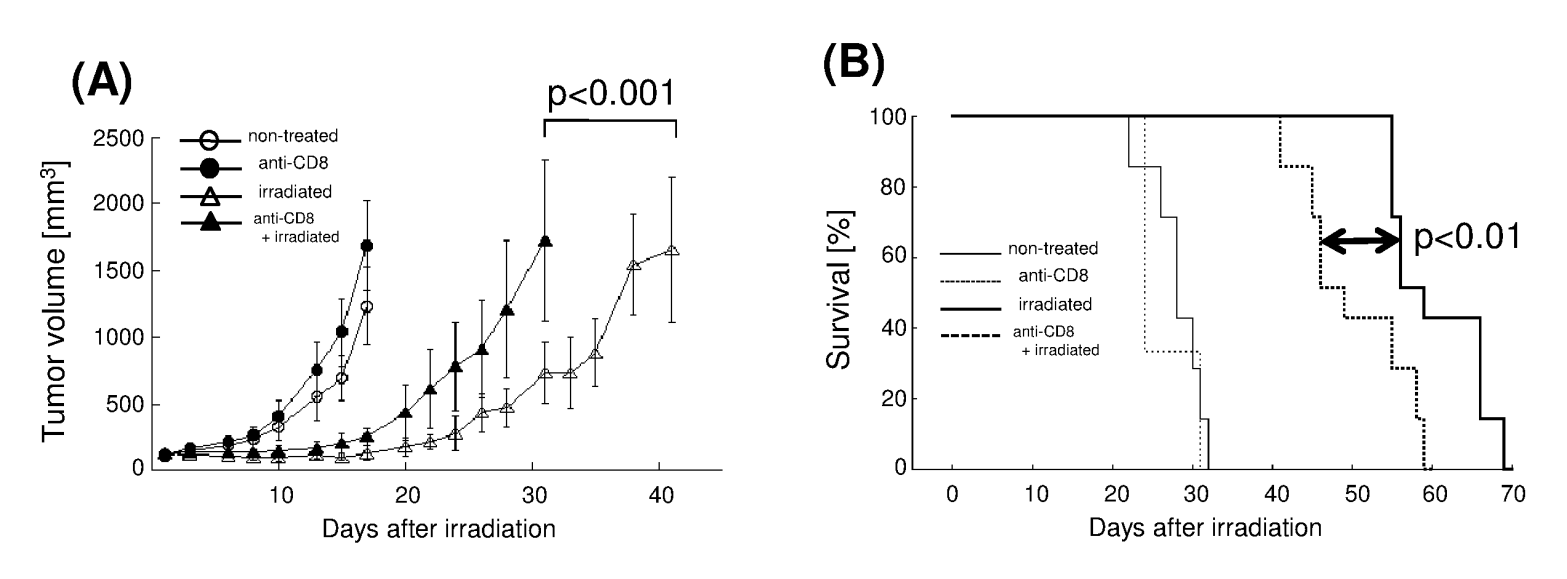
X-ray irradiation delays LL/C tumor growth, and CTL depletion reduces TGD, in C57BL/6 mice. (A) Growth curves of LL/C tumors in the non-treated group (open circles, n = 7), irradiated group (open triangles, n = 7), anti-CD8 antibody-treated group (closed circles, n = 3), and irradiation plus anti-CD8 antibody-treated group (closed triangles, n = 7). X-ray irradiation was performed when tumors reached 100 mm^3^ in volume (Day 0); bars, SD. (B) Survival curves for LL/C-inoculated C57BL/6 mice, non-treated group (thin solid line), irradiated group (thick solid line), anti-CD8 antibody-treated group (thin dotted line), and irradiation plus anti-CD8 antibody-treated group (thick dotted line).

Next, we evaluated tumor growth and survival times using an LL/C tumor model. Tumors were treated with 30 Gy of X-ray irradiation when they reached a volume of 100 mm^3^. Growth was significantly delayed by irradiation, with volumes reaching 500 mm^3^ at 28.7±2.2 days post-irradiation compared with 13.2±1.7 days in the non-irradiated group; hence the TGD was 15.4 days (Fig. 5A). To examine the involvement of immunity, we used a neutralizing anti-CD8 antibody. Depletion of CD8(+) cells using this antibody significantly decreased the TGD to 8.7±3.2 days (*p*<0.001; Fig. 5A). Median survival time (MST) in the anti-CD8 antibody plus irradiation group was 49 days (95% confidence interval [CI], 41.3–56.7), while that of the irradiation alone group was 59 days (95% CI, 51.3–66.7). Thus, CD8(+) cell depletion also significantly reduced survival time (*p*<0.01; Fig. 5B). Tumor growth and survival time were not markedly different between the anti-CD8 antibody alone group and the control group.

### CTLA-4 blockade enhanced the efficacy of irradiation in a mouse tumor model

**Figure 6:**
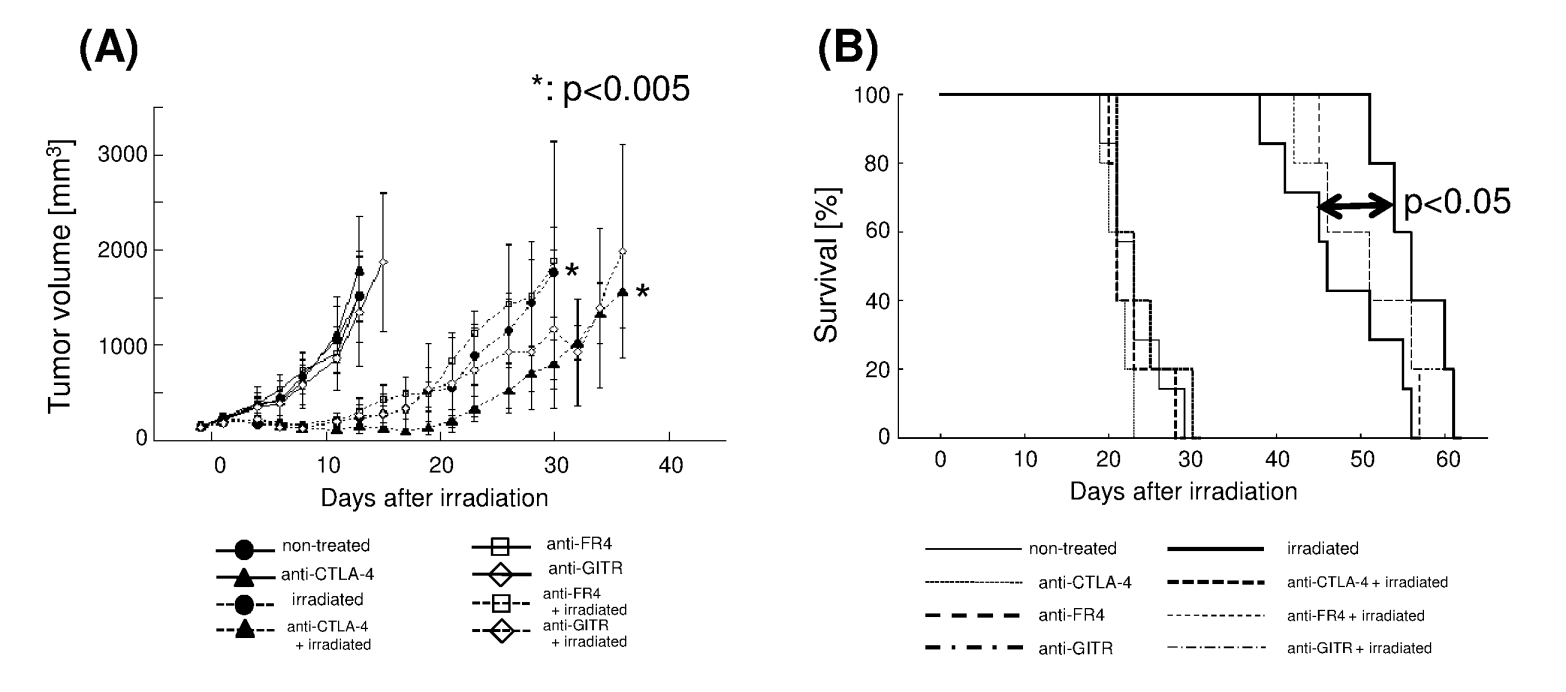
X-ray irradiation delays LL/C tumor growth and antibody-mediated immunomodulation increases TGD in C57BL/6 mice. (A) Growth curves of LL/C tumors in non-irradiated (solid line, n = 5/each group) and irradiated (dashed line, n = 5/each group) groups. Effects of the anti-CTLA-4 (closed triangles), anti-FR4 (open squares), anti-GITR (open diamonds) immunomodulatory antibodies, and the saline control (for the non-treated group, closed circles) were examined. X-ray irradiation was performed when tumors reached 100 mm^3^ in volume (Day 0); bars, SD. (B) Survival curves for each group of LL/C-inoculated C57BL/6 mice; non-treated (thin solid line), irradiated (thick solid line), anti-CTLA-4 antibody-treated (thin dotted line), irradiation plus anti-CTLA-4 antibody-treated (thick dotted line), anti-FR4 antibody-treated (thick dashed line), irradiation plus anti-FR4 antibody-treated (thin dashed line), anti-GITR antibody-treated (thick dash-dotted line), and irradiation plus anti-GITR antibody-treated (thin dash-dotted line).

We next examined whether an immunomodulatory antibody affected the efficacy of radiotherapy (Fig. 6A, 6B). Among the antibodies tested, an anti-CTLA-4 antibody significantly increased the anti-tumor efficacy of irradiation, with the TGD extended from 13.1±2.3 (irradiation alone group) to 19.5±2.9 (anti-CTLA-4 antibody plus irradiation group) days (*p*<0.005; Fig. 6A). Survival time was also longer in the anti-CTLA-4 antibody plus irradiation group (MST, 56 days; 95% CI, 51.7–60.3) than in the irradiation alone group (MST, 46 days; 95% CI, 43.4–48.6; *p*<0.05; Fig. 6B). Tumor growth and survival time were not markedly different between the anti-CTLA-4 antibody alone group and the control group (Fig. 6). The TGD and survival times of irradiated groups treated with anti-FR4 or anti-GITR antibodies did not differ significantly from those of the control group (Fig. 6). The average tumor volume tended to be slightly lower in the anti-GITR plus irradiation group than in the irradiation alone group; however, this was not statistically significant.

### X-ray irradiation induced secretion of HMGB1 protein from LL/C and EL4 cells *in vitro*

**Figure 7:**
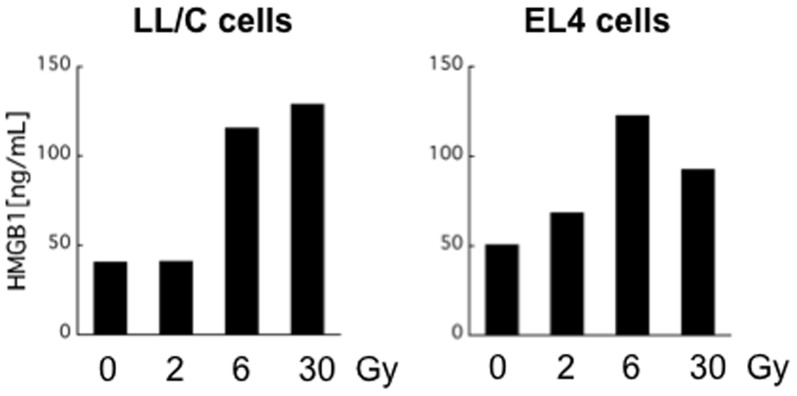
X-ray irradiation induces HMGB1 protein *in vitro.* Cells were exposed to the indicated doses of X-ray irradiation and then cultured for 48 h. HMGB1 concentrations in culture supernatants were measured by ELISA.

It was recently reported that X-ray irradiation-induced immunogenic cell death involves the secretion of HMGB1. Therefore, we examined whether X-ray irradiation induces HMGB1 release from LL/C and EL4 cells. Notably, HMGB1 was detected in the culture medium of irradiated LL/C cells 48 h after irradiation. Dose-dependent effects were observed: 2 Gy irradiation had no significant effect on HMGB1 release from LL/C cells, whereas higher doses (6 Gy and 30 Gy) induced greater amounts of HMGB1 (Figure 7). Similar results were obtained when EL4 cells were used (Figure 7). Thus, X-ray irradiation induces immunogenic cell death in these cells.

## Discussion

In the present study, we demonstrated that EL4 tumor-bearing mice acquired EL4-specific immunity after irradiation. Localized irradiation of tumors did not extend survival and did not induce EL4-specific immunity in nude mice. The role of the immune system was further confirmed using a LL/C tumor model. The efficacy of radiotherapy for treating LL/C tumors was reduced by depleting CD8(+) cells, with both TGD and survival time being significantly reduced. These results clearly indicate that CD8(+) cell activity influences the efficacy of radiotherapy. In addition, the efficacy of irradiation was augmented by anti-CTLA-4 antibody treatment.

Recent evidence suggests that anti-tumor immunity can be induced by radiotherapy. Furthermore, tumor-specific immune responses induced by irradiation play a crucial role in successful radiotherapy. Lee *et al*. recently reported that irradiation increases T cell priming, leading to tumor reduction in syngeneic models of B16 melanoma [8]. Takeshima *et al*. reported that CD8(+) cell depletion decreases the therapeutic efficacy of irradiation in a C57BL/6 mouse tumor model that used ovalbumin-transfected cells [9]. In the present study, C57BL/6 mice were inoculated with EL4 cells and then irradiated. Irradiation-induced anti-tumor immunity was confirmed by the following observations: (i) re-inoculated EL4 cells were completely rejected, (ii) EL4-specific cellular and humoral immunity was detected, and (iii) when mice were inoculated with tumors in both hind limbs, irradiation of one tumor caused shrinkage of the other, indicating an abscopal effect. We further quantified immune responses in an LL/C tumor model. Depleting CD8(+) cells using an anti-CD8 antibody significantly reduced the therapeutic efficacy of irradiation in terms of both tumor growth and survival time. These results demonstrate that local irradiation induces systemic tumor-specific immune responses, which are also essential for the local control of tumors. We emphasize that irradiation delayed tumor growth as a result of tumor-specific immune responses, not solely because of DNA breakage.

The results of this study, which demonstrate that activation of CD8(+) immune cells is involved in the efficacy of irradiation, suggest that therapeutic efficacy could be increased by augmenting the immune response. There are several approaches to activating anti-tumor immunity. The most promising procedure is immunomodulation using specific antibodies. Thus, we examined the effect of immunomodulatory antibodies on the efficacy of irradiation. Among the antibodies tested, an anti-CTLA-4 antibody increased the efficacy of irradiation, leading to increases in both TGD and survival time. CTLA-4 is expressed exclusively on T cells, where it primarily regulates the amplitude of the early stages of T cell activation. CTLA-4 blockade broadly enhances immune responses in animal models, including systemic immune hyperactivation and anti-tumor immunity. This increase in anti-tumor activity by CTLA-4 blockade is due to a combination of direct activation of effector T cell function and concomitant inhibition of regulatory T cell (Treg) activity [10]–[12]. Dewan *et al*. reported that the combined use of local irradiation and CTLA-4 blockade inhibits metastases in a mouse model of breast cancer [13]. The same group also reported that an anti-CTLA-4 antibody can induce an experimental abscopal effect in mouse models [14]. Taken together with our results, these findings indicate that irradiation-induced anti-tumor immunity can be augmented by CTLA-4 blockade and that the resulting enhanced immunity acts both locally and systemically. In initial studies, significant anti-tumor responses were observed in mice bearing immunogenic tumors; however, poorly immunogenic tumors did not respond to anti-CTLA-4 antibody monotherapy [10]. This is in agreement with our results demonstrating that the anti-tumor effect of the anti-CTLA-4 antibody was only observed when it was combined with irradiation. Thus, a combination of irradiation and anti-CTLA-4 antibody treatment may be an attractive approach. Clinically, a combination of local irradiation and anti-CTLA-4 antibody therapy caused regression of metastatic tumors at a distance from the irradiated site (abscopal effect) in a melanoma patient [2]. This tumor regression was associated with antibody responses against a tumor-specific cancer testis antigen, suggesting that augmentation of tumor-specific immune responses had occurred and demonstrating the promise of a combination of radiotherapy and CTLA-4 blockade in a clinical setting.

Several studies document tumor rejection following the depletion of Treg cells. Treg cells express high levels of FR4 and an anti-FR4 antibody specifically reduces Treg cell levels, thereby inducing effective anti-tumor immunity in tumor-bearing mice [15]. Treg cells also express high levels of GITR, and an agonistic antibody, DTA1, is an effective anti-tumor agent. When injected intratumorally, DTA1 exerts its anti-tumor activity by inhibiting the suppressor function of Treg cells [16], [17]. However, these two antibodies had no significant effect on the efficacy of irradiation in our investigation. One possible explanation is that the anti-CTLA-4 antibody inhibits Treg activity while also increasing the activity of effector cells such as CTLs, whereas the anti-FR4 antibody and DTA1 may not. Thus, in our *in vivo* model, it is possible that the anti-CTLA-4 antibody augmented both Treg depletion and effector cell activity. Another report noted that experimental outcomes can be influenced by the timing and dose of antibody administration [18]. Further studies are required to optimize the dose and timing of antibody administration when used in combination with irradiation.

In conclusion, this study demonstrates that tumor-specific immune responses play an important role in the therapeutic efficacy of irradiation, which can be augmented by immune checkpoint (CTLA-4) inhibition. The combination of radiotherapy and CTLA-4 blockade is a promising potential cancer therapy, although further study is required.

## Supporting Information

Figure S1 **Growth of EL4 tumor in unilateral and bilateral models.** (A) Timing of primary EL4 cell inoculation in unilateral and bilateral models. (B) Volume of tumors in right hind limbs of unilateral model mice (closed circles, n = 7), and tumors in right hind limbs of the bilateral model mice (open circles) and tumors of in left hind limb of the bilateral model mice (closed triangles, n = 7); bars, S.D.
(TIFF)
